# Hypervirulent *Clostridium difficile* ribotypes are CpG depleted

**DOI:** 10.1080/21505594.2018.1509669

**Published:** 2018-09-03

**Authors:** Vinay Kamuju, Santosh Kumar, Wajihul Hasan Khan, Perumal Vivekanandan

**Affiliations:** a Department of Biochemical Engineering and Biotechnology, Indian Institute of Technology Delhi, New Delhi, India; b Kusuma School of Biological Sciences, Indian Institute of Technology Delhi, New Delhi, India

**Keywords:** *Clostridium difficile*, hypervirulence, CpG depletion, dinucleotides, translational selection

**To The Editor**,

*Clostridium difficile* is emerging as a major enteric and nosocomial pathogen worldwide. Antibiotic usage is an important risk factor for *C.difficile* infection (CDI). The incidence of both nosocomial- and community-acquired CDI has been steadily increasing in the last 15 years. The increase in CDI rates has been primarily attributed to *C.difficile* PCR ribotype 027. Infection with *C.difficile* ribotype 027 has been associated with increased morbidity and mortality. Therefore, *C.difficile* ribotype 027 has been referred to as “hypervirulent”. The hypervirulence in ribotype 027 has been linked to a) increased transmissibility b) increased relapse rates and c) poor clinical outcomes as compared to typical endemic strains. In 2008, CDI with *C.difficile* PCR ribotype 078 was reported to cause severe disease. In addition, ribotypes 027 and 078 share virulence factors including *tcdA-, tcdB-* and binary toxin-genes [1]. Thus ribotypes 027 and 078 are both referred to as hypervirulent ribotypes of *C.difficile*. Although other ribotypes have been associated with epidemics and increased toxin production [2] there is no conclusive evidence of increased morbidity and mortality; therefore, the use of the term “hypervirulent” has thus far been limited to *C.difficile* ribotypes 027 and 078. In addition, differences in enzymes, expression of flagella, capsule production and enhanced adhesion have been attributed to hypervirulence in *C.difficile*. While a plethora of factors have been linked to ribotypes 027 and 078, it is accepted that hypervirulence is a complex process that is are not necessarily linked to toxin production, sporulation or resistance to antibiotics [3]. Despite extensive research, the specific genomic attributes of hypervirulence in *C.difficile* remain poorly understood.

Recent studies suggest that depletion of CpGs in virus genomes is linked to enhanced virus replication [4] and poor prognosis [5]. In addition, CpG dinucleotides content of virus genomes has been associated with virus pathogenesis [6], host methylation capabilities [7] and differences in evolutionary pressures [8]. Another recent report highlights how innate immune responses target non-self RNA in a CpG-dependent manner [9]. It is not known if the CpG content of bacterial genomes is associated with pathogenesis or virulence. Differences in CpG content of *C.difficile* strains has not been investigated as a potential factor in hypervirulence. We hypothesized that the CpG dinucleotide content in *C.difficile* is associated with hypervirulence.

A total of 21 whole genome sequences of *C.difficile* were retrieved from NCBI Genome database (https://www.ncbi.nlm.nih.gov/genome/genomes/535?) and analyzed. This includes all available full-length sequences from the hypervirulent ribotypes 027 and 078 (n = 15) and all available full-length sequences from the non-hypervirulent ribotype 012 (n = 6). Differences in transmission rates, ability to cause epidemics, toxin production, sporulation and antimicrobial resistance has been documented across *C.difficile* ribotypes [10,11]; nonetheless, only ribotypes 027 and 078 have been designated as “hypervirulent” based on several factors including increased morbidity and mortality associated with CDI by these ribotypes [1,12,13]. In addition, most studies investigating the mechanisms underlying hypervirulence use ribotype 012 as the reference, representing non-hypervirulent or “typical” strains. Therefore, for this study we considered ribotypes 027 and 078 as hypervirulent ribotypes. We used the historic ribotype 012 strain 630 [2] and all full-length sequences of ribotype 012 as “typical” or non-hypervirulent strains of *C.difficile*. The accession numbers of all the full-length *C.difficile* sequences analyzed are provided in Supplementary Table 1. The computation of dinucleotide ratio for double stranded *C.difficile* genome was carried out using the following formula [14]:

$$\left({{O \over E}} \right)XpY = \left({{O \over E}} \right)Y'pX' = {{2\left({fXpY + fY'pX'} \right)^*G} \over {\left({fX + fX'} \right)\left({fY + fY'} \right)}}$$

Where f(X), f(Y) and f(XpY) denote the mononucleotide frequencies and dinucleotide frequency respectively in one strand; f(X’), f(Y’) and f(Y’pX’) denote the frequency of complementary mononucleotides and reverse complement of the dinucleotide respectively in the same strand. G denotes the total length of genome.

Dinucleotide frequencies were calculated for all the whole genome sequences (n = 21). In addition, the dinucleotide frequencies in the coding DNA sequences (CDS) were calculated from the annotated Genbank files downloaded from NCBI (https://www.ncbi.nlm.nih.gov/sites/batchentrez). The calculation of dinucleotide frequencies for the coding regions was carried out for all annotated sequences [n = 12; this includes 8 sequences from hypervirulent ribotypes (027 and 078) and 4 sequences from ribotype 012]. Statistical analysis was carried out using the Mann Whitney U test, Student t test and Wilcoxon signed-rank test as appropriate. The bar graphs and box plots were made using the software Origin. The obtained results were considered significant at a p value <0.05.

**Figure 1.**
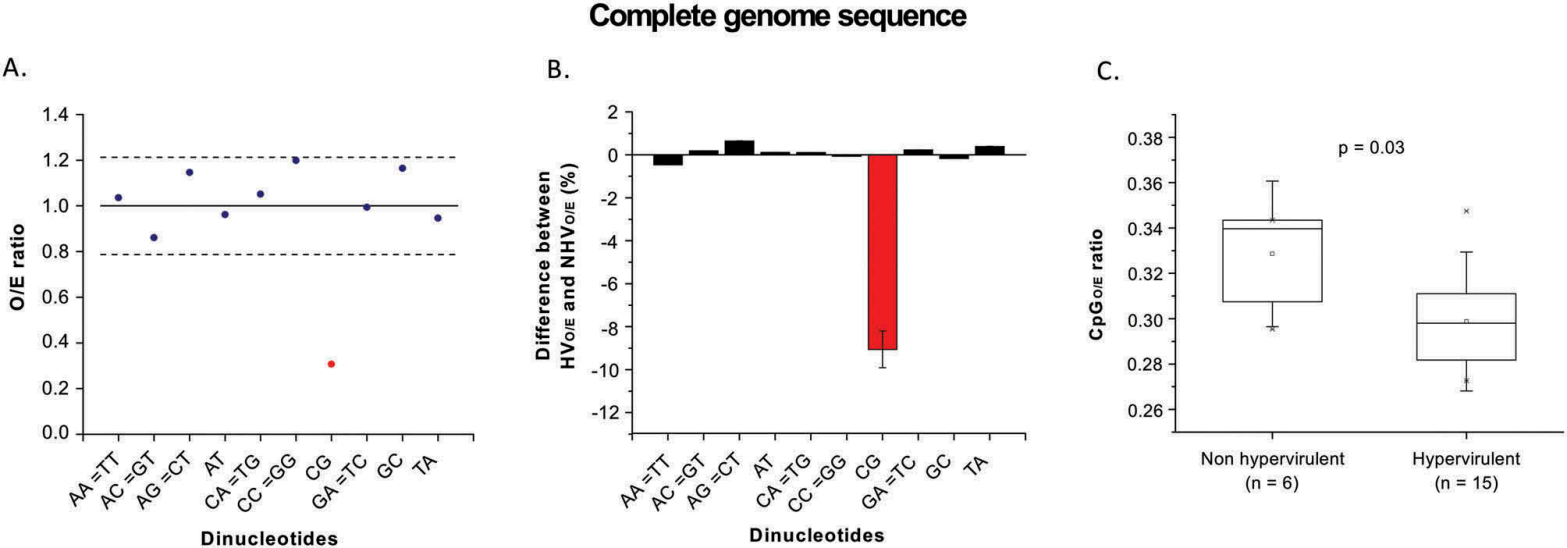
(a) CpG dinucleotides are depleted in *C.difficile*: Graph illustrating mean ± SD (the continuous black line indicates the mean and the broken black lines indicate standard deviation) of dinucleotide O/E ratio for *C.difficile* (both hypervirulent and non hypervirulent) (1 ± 0.213). All dinucleotides except the CpG dinucleotides (red) lie within the confidence interval of (0.787–1.213). (b) CpG dinucleotides are the most variable dinucleotides between hypervirulent and non-hypervirulent *C.difficile* strains: The difference in dinucleotide O/E ratios between hypervirulent (HV) and non-hypervirulent (NHV) *C.difficile* strains in percentage is shown in the Y-axis (A positive value for a given dinucleotide implies higher O/E ratios for the hypervirulent strains as compared to the non-hypervirulent strains). The complete genome sequences were analyzed. The differences between the hypervirulent and the non-hypervirulent strains were less than 1% except for the CpG dinucleotide for which the difference is about 9% (red bar). (c) Hypervirulent *C.difficile* strains are more CpG depleted than the non-hypervirulent strains: Box-plot diagram showing the CpG (O/E) in the whole genome sequence in non-hypervirulent and hypervirulent strains of *C.difficile*. The relative abundance of CpG dinucleotides was significantly higher in non-hypervirulent strains (Median = 0.341) as compared to the hypervirulent strains (Median = 0.298) [p < 0.05].

We found that CpG dinucleotides were the most depleted dinucleotides among the *C.difficile* strains analyzed (i.e. both hyper-virulent and non-hypervirulent ribotypes). The relative abundance of CpG dinucleotides in *C.difficile* strains analyzed is 0.307 (Figure 1(a); i.e. only about 30% of the expected number of CpGs are present). Interestingly, CpG dinucleotides were the most variable dinucleotides (about 9% for CpG vs about 1% for other dinucleotides) between the hypervirulent and the non-hypervirulent ribotypes of *C.difficile* (Figure 1(b)). Importantly, the hypervirulent ribotypes of *C.difficile* had significantly lower CpG content as compared to the non-hypervirulent ribotype (Figure 1(c); p value = 0.03). These findings suggest that CpG is a rapidly evolving dinucleotide in *C.difficile* genomes and also highlight the potential link between depletion of CpG content and hypervirulence in *C.difficile*. Given that higher CpG content in bacterial genomes has been linked with enhanced activation of the toll-like receptor 9 (TLR-9) [15], our finding of higher CpG content in non-hypervirulent ribotypes of *C.difficile* as compared to hypervirulent ribotypes is particularly interestingly. Furthermore, *C.difficile* encoded toxins have been shown to bind to *C.difficile* genomic DNA with high affinity leading to activation of TLR-9 responses [16]. Therefore, our finding of CpG depletion from hypervirulent *C.difficile* ribotypes may be potentially associated with weak TLR-9 responses.

**Figure 2.**
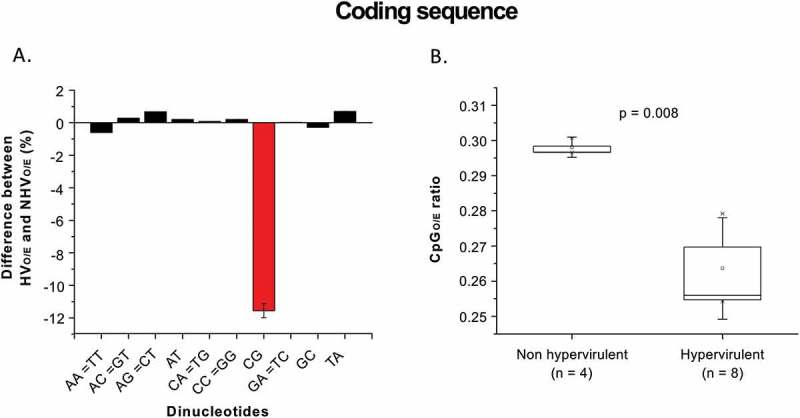
(a) Within the coding regions CpG dinucleotides are the most variable dinucleotides between hypervirulent and non-hypervirulent *C.difficile* strains: The difference in dinucleotide O/E ratios between hypervirulent (HV) and non-hypervirulent (NHV) *C.difficile* strains within the coding region (in percentage) is shown in the Y-axis (A positive value for a given dinucleotide implies higher O/E ratios for the hypervirulent strains as compared to the non-hypervirulent strains). The differences between the hypervirulent and the non-hypervirulent strains were less than 1% except for the CpG dinucleotide for which the difference is about 11.5% (red bar). (b) Within the coding region CpG depletion is more pronounced in the hypervirulent *C.difficile* strains as compared to the non-hypervirulent strains: Box-plot diagram showing the CpG (O/E) in the coding region in non-hypervirulent and hypervirulent strains of *C.difficile*. The relative abundance of CpG dinucleotides was significantly lower in the hypervirulent strains (Median = 0.263) as compared to the non-hypervirulent strains (Median = 0.298) [p < 0.05].

Further analysis of the coding regions suggests that the differences between hypervirulent and non-hypervirulent ribotypes for all other dinucleotides (except CpG dinucleotides) were marginal (<1% for all other dinucleotides as compared to 11.5% for CpG dinucleotides; Figure 2(a)). Importantly, the CpG dinucleotide depletion in the hypervirulent ribotypes is more pronounced in the coding DNA sequences (CDS) compared to the whole genome (0.263 in the CDS vs 0.298 in the whole genome; Figures 2(b) and 1(c); p value = 0.008). The differences in the CpG content (both at the whole genome level and within the CDS) between hypervirulent and non-hypervirulent ribotypes is more pronounced as compared to that of other dinucleotides. Our results suggest a potential role for CpG depletion in the hypervirulence of *C.difficile* although we do not identify specific underlying mechanisms. To the best of our knowledge, this is the first report, linking CpG depletion in bacterial genomes to virulence.

As with eukaryotic genomes, methylation of CpGs in bacterial genomes has been linked to the depletion of CpGs [17]. If the depletion of CpG dinucleotides from the hypervirulent ribotypes of *C.difficile* is due to mutational pressure, we would expect that the loss of CpGs from the whole genome is more pronounced than that from the coding. The increased depletion of CpG dinucleotides from the coding regions of the hypervirulent ribotypes suggests a major role for translational selection (i.e. codon usage bias); nonetheless we cannot rule out a role for mutational pressure. A recent report has documented CpG depletion in bacterial genomes [17]; this report also suggests a potential link between CpG-specific DNA methyltransferases encoded by bacteria and the loss of CpG dinucleotides. In addition, cytosine methylation in bacterial genomes has been associated with modulation of gene expression, motility, adhesion and virulence [18]. Although CpG methylation is well-documented in bacteria, the role of CpG depletion in bacterial pathogenesis has not been investigated.

Hypervirulence in *C.difficile* has been linked to adaptation to the host [19] and differences in gene expression [20]. In our study, we found that transcripts encoded by hypervirulent *C.difficile* ribotypes contain significantly lower number of CpGs than those encoded by the non-hypervirulent ribotypes. The reduced CpG content in transcripts of hypervirulent *C.difficile* ribotypes may be associated with increased translation efficiencies or escape from innate immune responses such as ZAP that targets CpG-containing mRNA.

In sum, our results suggest that (a) *C.difficile* genomes are CpG depleted (b) CpG dinucleotides are the most rapidly evolving dinucleotides in *C.difficile* and (c) Hypervirulent *C.difficile* ribotypes are significantly CpG depleted (both at the whole genome level and within coding regions) as compared to the non-hypervirulent strains. This work highlights previously unknown differences between hypervirulent and non-hypervirulent ribotypes of *C.difficile*. Importantly, these results provide a novel perspective on hypervirulence in *C.difficile* and lay the groundwork for studies investigating systematic CpG depletion in bacterial pathogenesis.
