# Supplementary material for: Hypervirulent Clostridium difficile ribotypes are CpG depleted
Source: Virulence. 2018 Sep 3;9(1):1422–5. doi: 10.1080/21505594.2018.1509669 (PMC6141142; doi:10.1080/21505594.2018.1509669)
Supplement: Supplemental Material [file kvir-09-01-1509669-s001.docx]

**Table 1:** List of *Clostridium difficile* strains used in this study

| **Classification** | **Accession number** | **Strain name** | **Ribotype** |
| --- | --- | --- | --- |
| **Hypervirulent (n=15)** | FN665654.1 | 2007855 | 27 |
|  | FN668941.1 | BI1 | 27 |
|  | NC_013315 | CD196 | 27 |
|  | NZ_CM000659 | CIP10792 | 27 |
|  | NZ_CM000660 | QCD-23m63 | 27 |
|  | CM000287.4 | QCD-32g58 | 27 |
|  | NZ_CM000658 | QCD-37x79 | 27 |
|  | NZ_CM000441 | QCD-66c26 | 27 |
|  | NZ_CM000661 | QCD-76w55 | 27 |
|  | NZ_CM000657 | QCD-97b34 | 27 |
|  | FN545816.1 | R20291 | 27 |
|  | NC_017174 | M120 | 78 |
|  | ADVM00000000.1 (WGS scaffold) ADVM01000001:ADVM01000100 (contigs) | NAP07 | 78 |
|  | ADNX00000000.1 (WGS scaffold) ADNX01000001:ADNX01000111 (contigs) | NAP08 | 78 |
|  | CAMC00000000.1 (WGS scaffold) CAMC01000001:CAMC01000210 (contigs) | T20 | 78 |
| **Non hypervirulent (n= 6)** | NC_009089 | C 630 | 12 |
|  | NZ_CP010905 | C 630 | 12 |
|  | NZ_CP016318 | C630 DERM | 12 |
|  | NZ_LN614756 | 630 delta erm | 12 |
|  | CAMX00000000.1(WGS scaffold) CAMX01000001-CAMX01000274 (contigs) | E28 | 12 |
|  | CAMW00000000.1(WGS scaffold) CAMW01000001-CAMW01000275 (contigs) | T3 | 12 |
